# Role of the glutamatergic system of ventrolateral periaqueductal gray (vlPAG) in the cardiovascular responses in normal and hemorrhagic conditions in rats

**DOI:** 10.22038/ijbms.2021.53181.11978

**Published:** 2021-05

**Authors:** Vida Alikhani, Reza Mohebbati, Mahmoud Hosseini, Abolfazl Khajavirad, Mohammad Naser Shafei

**Affiliations:** 1 Department of Physiology, Faculty of Medicine, Mashhad University of Medical Sciences, Mashhad, Iran; 2 Applied Biomedical Research Center, Mashhad University of Medical Sciences, Mashhad, Iran; 3 Division of Neurocognitive Sciences, Psychiatry and Behavioral Sciences Research Center, Mashhad University of Medical Sciences, Mashhad, Iran

**Keywords:** Blood pressure, Cardiovascular system, Glutamic acid, Hemorrhage, Periaqueductal gray

## Abstract

**Objective(s):**

Periaqueductal gray (PAG) is a mesencephalic area divided into four columns including ventrolateral periaqueductal gray (vlPAG). vlPAG plays a role in cardiovascular regulation during normal and hemorrhagic (Hem) conditions. Due to presence of glutamate in this area, we evaluated the effect of glutamatergic receptors of this area on cardiovascular activity in normotensive and hypovolemic Hem rats.

**Materials and Methods:**

Animals were divided into twelve groups: saline (vehicle), Glutamate, GYK52466 (non-NMDA receptor antagonist), and MK801 (NMDA receptor antagonist) with and without Glu microinjected into vlPAG in normal and Hem conditions. Following the femoral artery cannulating and microinjecting, changes (Δ) of heart rate (HR), systolic blood pressure (SBP), and mean arterial pressure (MAP) were recorded via a PowerLab unit.

**Results:**

In normotensive conditions, microinjection of Glu increased ΔMAP, ΔSBP, and ΔHR (*P*<0.001). MK-801 and GYKI-52466 nonsignificant reduced cardiovascular responses than vehicle while their changes were significant compared with glutamate (*P*<0.001). Co-injection of GYKI- 52466 with Glu did not significantly reduce ΔSBP and ΔMAP induced by Glu (*P*>0.05) but co-injection of MK-801 with Glu significantly attenuate these effects(*P*<0.01). In Hem, Glu increased ΔSBP, ΔMAP, and ΔHR (*P*<0.05). GYKI-52466 alone did not change cardiovascular responses but MK-801 decreased ΔSBP than Hem (*P*<0.01). Co-injection of GYKI-52466 with Glu had significant(*P*<0.05) but MK-801 with Glu had no significant effect compared with Hem (*P*>0.05).

**Conclusion:**

The glutamatergic system of vlPAG increases cardiovascular values that are mostly mediated through the NMDA receptor. Since vlPAG is well known as an inhibitory region, it seems that glutamate does not have a noteworthy cardiovascular role in vlPAG during Hem and normal conditions.

## Introduction

Periaqueductal gray (PAG) is a mesencephalic structure described as being related to the neuronal pathways that affect cardiovascular regulation and autonomic function (1). PAG divides into four longitudinal columns anatomically: dorsomedial (dmPAG), dorsolateral (dl PAG), lateral (lPAG), and ventrolateral (vlPAG) columns (2). VlPAG has various functions including, fear and defensive behavior (3), food intake (4), rapid eye movement (REM), sleep regulation (5), and involvement in pain modulation (6). VlPAG projects to numerous cardiovascular regulating areas such as the rostral ventromedial medulla (RVLM), an important cardiovascular regulation region. (7). Some studies show that chemical stimulation of vlPAG decreases blood pressure (BP) and heart rate (HR) in rats (1, 7). VlPAG is also related to the nucleus tractus solitarius (NTS), a crucial area for baroreflex and chemoreflex control. There is evidence that vlPAG could modulate tachycardia followed by baroreflex activation (1, 8). 

Various neurotransmitters such as acetylcholine, norepinephrine, and glutamate (Glu) are found in vlPAG, which cause different responses in BP and HR (1). It has been reported that vlPAG neurons are activated during hemorrhage, and inactivation of vlPAG can cause hypotension and hemorrhagic bradycardia (9). Glutamate through metabotropic and ionotropic (divide into N-methyl-d-aspartic acid (NMDA) and non- N-methyl-d-aspartic acid (non-NMDA) receptor subtypes) receptors plays a crucial role in regulating the cardiovascular central system (10). Some studies depict the role of glutamate receptors in hemorrhage and blood pressure (11-13). Furthermore, Glu and its ionotropic receptors (NMDA and non-NMDA) in vlPAG nucleus have been reported (11, 14). The blockade of NMDA glutamate receptor in vlPAG inhibits cardiovascular responses induced by the lateral hypothalamic area (11, 15). The aim and novelty of the current study is evaluation of the Glut receptor types involved in the cardiovascular responses during normal and hemorrhage conditions in vlPAG nucleus. Also, in this study, it is significant whether excitation of Glut projection from vlPAG to RVLM directly or indirectly has a role in improvement of HEM condition.

## Materials and Methods

***Animals***

Seventy-two male Wistar rats (250–290 g) were delivered from the animal house of the Medicine Faculty at Mashhad University of Medical Sciences. The rats were housed under standard conditions with *ad libitum* feeding and water, under a 12-hr light/dark cycle. The experimental process was conducted in accordance with the University Ethical Committee guidelines (approval ID: IR.MUMS.MEDICAL.REC.1398.338).

***Drugs***

Glutamate (Glu), the principal excitatory neurotransmitter in the central nervous system, GYKI-52466 (1-(4-aminophenyl)-4 methyl-7, 8-methylenedioxy-5H-2, 3-benzodiazepine) (GYK, a selective non-competitive AMPA (non-NMDA) receptor antagonist), MK-801 (MK, a selective non-competitive NMDA antagonist), and urethane, as an anesthetic, were used in this study (16, 17). All drugs were purchased from Sigma Aldrich Chemical Co., USA. 

***Animal cannulation and cardiovascular response measurement***

At first, the rats were anesthetized deeply with urethane (1.5 g/kg). The femoral artery was then cannulated with a heparinized angiocath catheter (22-gauge) for recording cardiovascular parameters and withdrawing blood by connected syringe (18). The angiocath catheter was connected to a blood pressure transducer attached to a PowerLab system (ID instrument, Australia). BP and HR were recorded by the PowerLab system. 

During the study, the animal’s body temperature was maintained at 37.5 °C with a warmer throughout the experiment.

***Stereotaxic and drug microinjection***

After arterial cannulation, the animal was mounted on the stereotactic frame, and the head was fixed. VlPAG area’s coordination was determined based on Paxinos and Watson rat brain atlas (AP: 6.6–8.7 mm, L: ±0–1.5 and H: 5.5–6.5 mm) (19). Then, a hole about 2 mm in diameter was drilled into the skull, and drugs were microinjected into vlPAG using a micropipette with 35–40 µm diameter (Stoelting, USA) connected to a syringe and attached to a manual injector (Stoelting, USA) (20). 

***Animals groups***

The animals were divided into two main groups, including (A) normotensive and (B) hypotensive hemorrhagic (Hem), then subdivided into the following subgroups (n=6): 

A) Normotensive groups: 1) vehicle (saline), 2) Glu, 3) GYK, 4) Co-injection of GYK + Glu, 5) MK, and 6) Co-injection of MK + Glu 

B) Hemorrhage (Hem) groups: 1) vehicle, 2) Glu, 3) GYK, 4) GYK + Glu, 5) MK, and 6) MK + Glu were microinjected into vlPAG.

Doses of drugs in all groups for Glu, GYK, and MK were 50 nmol, 300 nmol, and 0.5 nmol, respectively (21-23). The microinjection volume for all drugs was 100–150 nl (18).

***Hemorrhage protocol***

In Hem groups, after stabilization of cardiovascular parameters (approximately 5 min), about 15% of Total Blood Volume (TBV) was withdrawn during ten minutes (5^th^ min to 15^th^ min) from the femoral artery cannula (18). Hem was induced before microinjection. TBV was calculated according to this equation: 0.06 ml per gr (Body Weight)× Body Weight +0.77 (24). This volume (15%) could reduce about 30 mmHg of SBP that appropriate conditions to assess central cardiovascular areas involved in Hem (24). At the end of the experiment, animals were sacrificed by an overdose of urethane. The brains were removed from skulls and kept for 24 hr in 10% formalin for tissue fixation; next, a vibratome was used to cut thin slices with 60-micron thickness. The slides were observed under a light microscope for verification of the microinjection site, according to atlas of Paxinos and Watson (25). 

***Data analysis***

The data were expressed as mean±SEM. Cardiovascular variables, including MAP, HR, and SBP were recorded, and their changes (Δ) were calculated to evaluate the trend of changes several times. Analysis of this data was done by repeated-measures ANOVA, followed by a *post-hoc* Tukey’s test. Moreover, peak changes of ΔSBP, ΔMAP, and ΔHR were analyzed using (one-way ANOVA and Tukey’s *post hoc *test). *P*<0.05 was considered significant.

## Results

***Effect of saline microinjected into vlPAG nucleus on cardiovascular responses in normotensive rats***

In this group, cardiovascular responses before and after microinjection of saline were examined. Before saline microinjection, the cardiovascular responses for MAP, SBP, and HR were 113.34±12.5 mmHg, 135.9 ± 11.16 mmHg, and 384.8 ± 17.22 beats/min, respectively. However, microinjection of saline did not significantly change those parameters (MAP: 110.4±10.6 mmHg, SBP: 131.3±9.2 mmHg, and HR: 379.5 ± 14.6 beats/min).

***Effect of glutamate, GYK, and MK microinjected into vlPAG nucleus on cardiovascular responses in normotensive rats***

**Figure 1:**
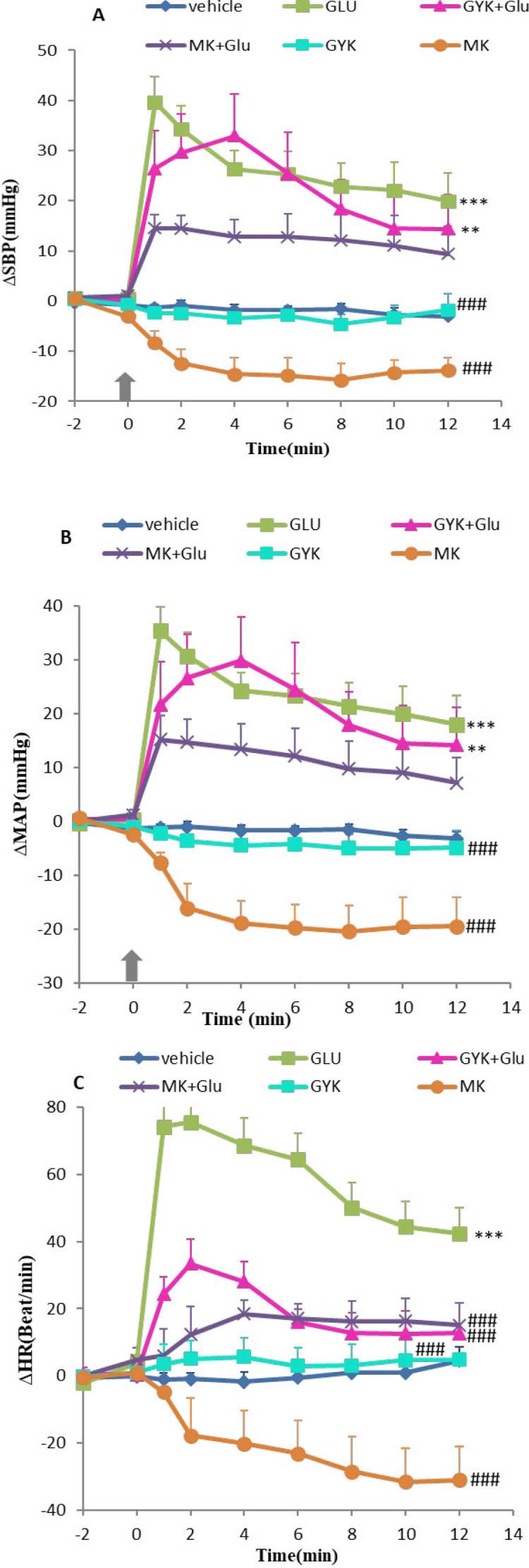
Time course of ∆SBP (A), ∆MAP(B), and ∆HR (C), after microinjection of saline, glutamate, and NMDA (MK) and non-NMDA antagonist (GYK) of glutamate receptor into vlPAG nucleus. Data were expressed as mean±SEM; n= 6 (repeated measures ANOVA). ∆MAP: mean arterial pressure, ∆SBP: systolic blood pressure, ∆HR: heart rate, vehicle: saline microinjection, Glu: glutamate, GYK: GYKI-52466, MK: MK801. ***: *P*<0.001, **: *P*<0.01, and *: *P*<0.05 vs vehicle group, ###:*P*<0.001, ##: *P*<0.01, and #: *P*<0.05 vs Glu group

To determine the cardiovascular effects, Glu, GYK, and MK were microinjected into vlPAG, and cardiovascular changes were evaluated. Microinjection of Glu alone increased ∆SBP, ∆MAP, and ∆HR compared with the vehicle group (*P*<0.001, Figure 1 parts A, B, and C, respectively). Microinjection of GYK and MK alone did not change the cardiovascular parameters compared with the vehicle group over time (repeated measures ANOVA, *P*>0.05) while their co-injections with Glu attenuated the Glu response. ∆SBP and ∆MAP in GYK + Glu showed significant differences compared with the vehicle group (*P*<0.01). However, the effect of MK + Glu on cardiovascular parameters did not significantly change (*P*>0.05) compared with the vehicle group. 

Time-course changes of ∆SBP, ∆MAP, and ∆HR in Glu, GYK, and MK groups have also been shown in Figure 1. The difference of GYK and MK in ∆SBP, ∆MAP, and ∆HR was significant compared with the Glu group over time (repeated measures ANOVA, *P*<0.001, Figure 1). ∆SBP and ∆MAP differences in co-injection of GYK + Glu and MK + Glu groups did not significantly change (*P*>0.05, Figures 1 A and B), and only ∆HR decreased significantly compared with the Glu group (*P*<0.001, Figure 1 C).

**Figure 2:**
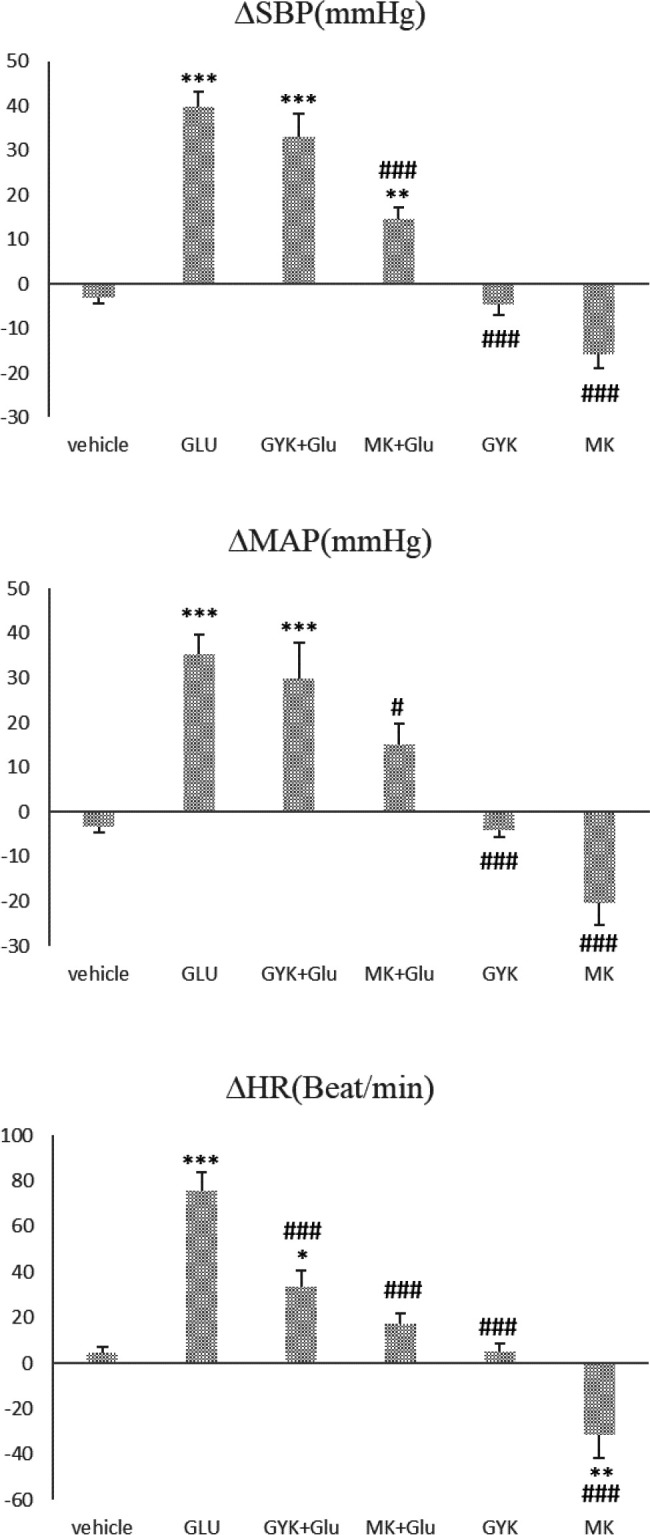
Peak changes of ∆SBP (a), ∆MAP(b), and ∆HR (c), after microinjection of saline, glutamate, NMDA (MK), and non-NMDA antagonist (GYK) of glutamate receptor and their co-injection with Glu into vlPAG nucleus. Data were expressed as mean±SEM; n= 6 (one-way ANOVA). ∆MAP: mean arterial pressure, ∆SBP: systolic blood pressure, ∆HR: heart rate, vehicle: saline microinjection, Glu: glutamate, GYK: GYKI-52466, MK: MK801. ***: *P*<0.001, **: *P*<0.01, and *: *P*<0.05 vs vehicle group, ###: *P*<0.001, ##: *P*<0.01, and #: *P*<0.05 vs Glu group

Glu microinjection into vlPAG significantly increased all cardiovascular parameters’ peak changes compared with the vehicle group (*P*<0.001, Figure 2). Also, GYK + Glu enhanced the peak change of ∆SBP, ∆MAP, and ∆HR compared with the vehicle group (*P*<0.05 to *P*<0.001, Figure 2). MK + Glu microinjection increased ∆SBP (*P*<0.01, Figure 2 A), and MK alone decreased the peak changes in ∆HR compared with the vehicle group (*P*<0.01, Figure 2 C).

GYK and MK microinjection alone and co-injection of MK + Glu significantly decreased the peak changes of vascular parameters compared with the Glu group (*P*<0.05 to *P*<0.001, Figure 2), and co-injection of GYK + Glu just decreased the peak change of ∆HR compared with the Glu group (*P*<0.001, Figure 2 C).

***Effect of glutamate, GYK, and MK microinjected into vlPAG nucleus on cardiovascular responses in hemorrhagic rats***

**Figure 3:**
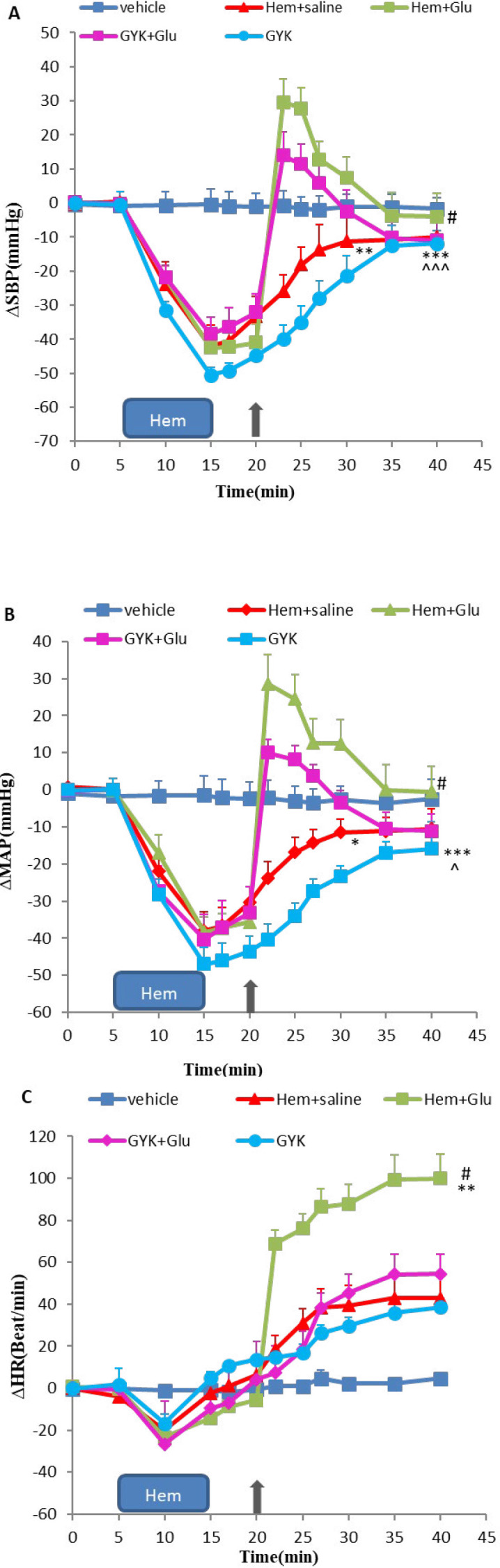
Time course of ∆SBP (A), ∆MAP(B), and ∆HR (C) after microinjection of saline, glutamate, GYK the non-NMDA antagonist of glutamate receptor, and co-injection of GYK and Glu into vlPAG nucleus in hemorrhagic condition. Data were expressed as mean±SEM; n= 6 (repeated measures ANOVA). ∆MAP: Mean arterial pressure, ∆SBP: Systolic blood pressure, ∆HR: Heart rate, vehicle: saline microinjection, Glu: glutamate, GYK: GYKI-52466. Differences with *P*-value <0.05 were considered significant. ***: *P*<0.001, **: *P*<0.01, and *: *P*<0.05 vs vehicle group, ###: *P*<0.001, ##: *P*<0.01, and #: *P*<0.05 vs Hem+saline group and ^^^: *P*<0.001, ^^: *P*<0.01, and ^: *P*<0.05 vs Glu group

**Figure 4:**
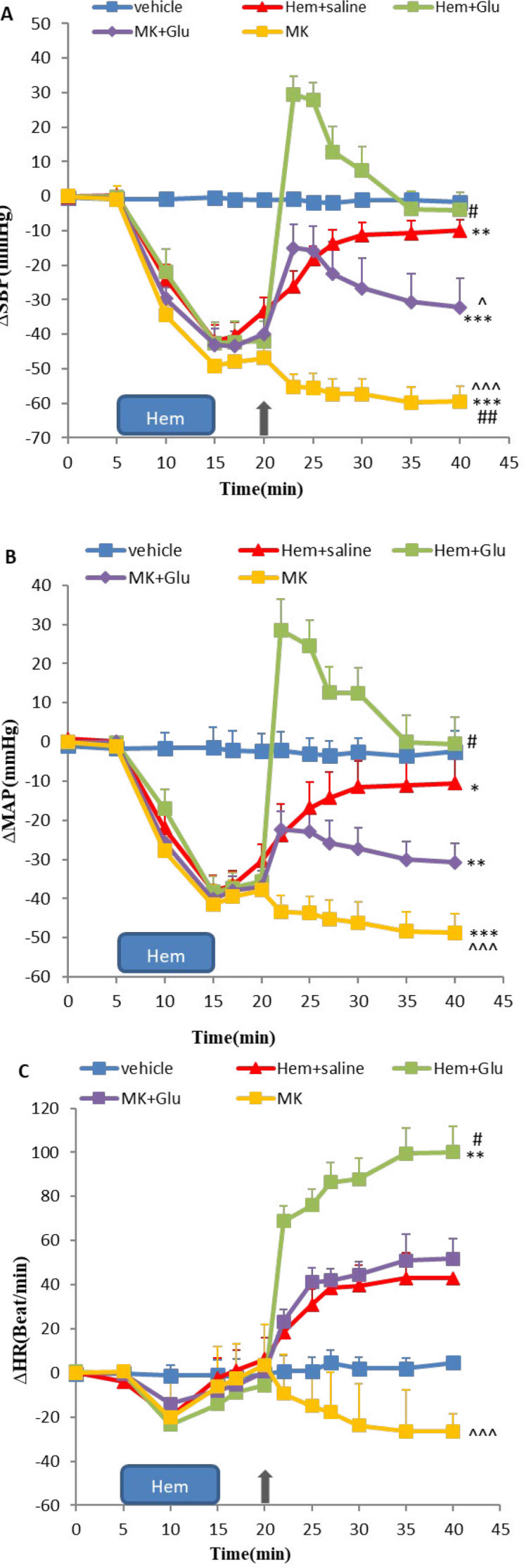
Time course of ∆SBP (A), ∆MAP(B), and ∆HR (C), after microinjection of the saline, glutamate, MK the NMDA antagonist of glutamate receptor, and co-injection of MK and Glu into vlPAG nucleus in hemorrhagic condition. Data were expressed as mean±SEM; n= 6 (repeated measures ANOVA). ∆MAP: mean arterial pressure, ∆SBP: systolic blood pressure, ∆HR: heart rate, vehicle: saline microinjection, Glu: glutamate, GYK: GYKI-52466, MK: MK801. Differences with *P*-value <0.05 were considered significant. ***: *P*<0.001, **: *P*<0.01, and *: *P*<0.05 vs vehicle group, ###: *P*<0.001, ##: *P*<0.01, and #: *P*<0.05 vs Hem+saline group and ^^^: *P*<0.001, ^^: *P*<0.01, and ^: *P*<0.05 vs Glu group

In the current experiment, to investigate the role of glutamatergic neurons of vlPAG in hypovolemic hypotension condition, 5 min after Hem, Glu, GYK, and MK alone and together were microinjected into vlPAG, cardiovascular responses were evaluated. Time-course changes of ∆SBP, ∆MAP, and ∆HR in the Hem groups treated with Glu, GYK, and MK are shown in Figures 3 and 4, separately. As it has been indicated, Hem induction caused a significant decrease in ∆SBP and ∆MAP compared with the vehicle group over time (repeated measures ANOVA, *P*<0.05 to *P*<0.01), and ∆HR increased, but it was not significant (*P*>0.05). Microinjection of Glu into vlPAG ameliorates the hypotensive responses induced by Hem over time (repeated measures ANOVA, *P*<0.05, Figure 3). ∆SBP, ∆MAP, and ∆HR induced by Hem did not change after microinjection of GYK alone and co-injection with Glu compared (*P*>0.05, Figure 3). ∆SBP changes in the MK alone group significantly decreased compared with the Hem group (*P*<0.01, Figure 4 A). Co-injection of MK + Glu did not change the cardiovascular responses compared with the Hem group (*P*>0.05, Figure 4).

Co-injection of MK + Glu significantly reduced ∆SBP and ∆MAP with respect to the Glu group over time (repeated measures ANOVA, *P*<0.05 to *P*<0.01, Figures 4 A and B). GYK + Glu effects on ∆SBP, ∆MAP, and ∆HR compared with the Glu group were not significant (*P*>0.05, Figure 3). GYK microinjection decreased ∆SBP and ∆MAP compared with the Glu group over time (repeated measures ANOVA, *P*<0.05 to *P*<0.001 Figures 3 A and B), and MK microinjection significantly decreased the cardiovascular responses compared with the Glu group (*P*<0.001, Figures 3 and 4, parts A, B, and C).

**Figure 5:**
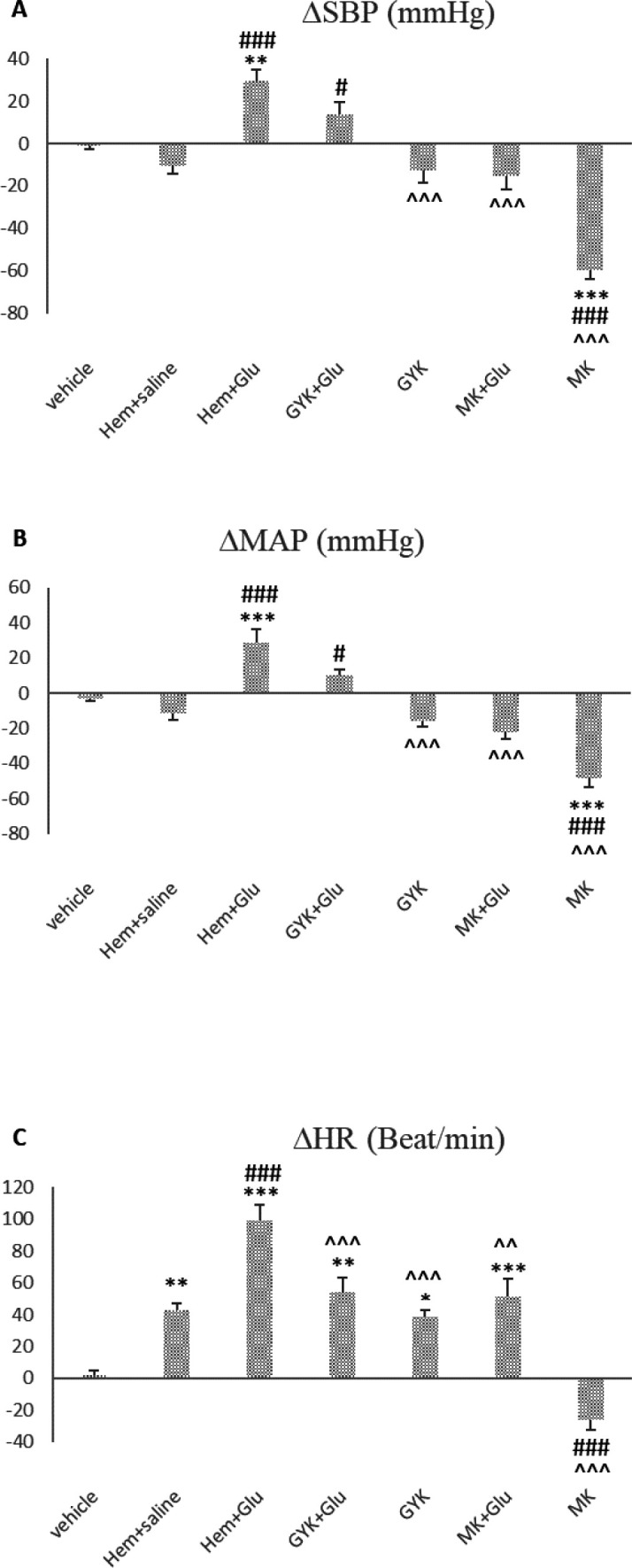
Peak changes of ∆SBP (A), ∆MAP (B), and ∆HR (C) after microinjection of saline, glutamate, NMDA (MK), and non-NMDA antagonist (GYK) of glutamate receptor and co-injection of them with Glu into vlPAG nucleus in hemorrhagic condition. Data were expressed as mean±SEM; n=6 (one-way ANOVA). ∆MAP: mean arterial pressure, ∆SBP: systolic blood pressure, ∆HR: heart rate, vehicle: saline microinjection, Glu: glutamate, GYK: GYKI-52466, MK: MK801. Differences with *P*-value<0.05 were considered significant. ***: *P*<0.001, **: *P*<0.01, and *: *P*<0.05 vs vehicle group, ###: *P*<0.001, ##: *P*<0.01, and #: *P*<0.05 vs Hem+saline group and ^^^: *P*<0.001, ^^: *P*<0.01, and ^: *P*<0.05 vs Glu group

Hem significantly increased the peak changes of ∆HR compared with the vehicle group (*P*<0.01 Figure 5 C). The peak change of ∆SBP and ∆MAP non-significantly decreased (*P*>0.05, Figures 5 A and B). Glu microinjection increased the peak changes of ∆SBP, ∆MAP, and ∆HR compared with the vehicle group (*P*<0.01 to *P*<0.001, Figure 5).

The peak changes of the vascular parameters showed that hypotension induced by Hem improved by microinjection of Glu (*P*<0.001, Figure 5), and the peak changes of ∆MAP and ∆SBP were ameliorated by co-injection of GYK + Glu (*P*<0.05), with no significant effect on ∆HR (*P*>0.05, Figure 5). None the peak changes of vascular parameters were affected by GYK alone (*P*>0.05, Figure 5) compared with the Hem group. The peak changes of ∆SBP, ∆MAP, and ∆HR were significantly changed by MK (*P*<0.001, Figure 5), but MK + Glu’s co-injection did not alter the peak changes of ∆SBP, ∆MAP, and ∆HR in comparison with the Hem group (*P*>0.05).

The peak changes of ∆SBP, ∆MAP, and ∆HR in GYK, MK, and MK + Glu groups significantly decreased compared with the Glu group (*P*<0.01 to *P*<0.001, Figure 5). Co-injection of GYK + Glu did not cause a significant difference in ∆SBP and ∆MAP compared with the Glu group (*P*>0.05), and only peak changes of ∆HR significantly decreased (*P*<0.001, Figure 5 part C).

## Discussion

According to the present study, in normotensive rats, microinjection of Glu into vlPAG increased cardiovascular responses, and these effects were mostly mediated by the NMDA receptor, while non-NMDA antagonists did not affect cardiovascular responses. Co-injection of Glu with NMDA and non-NMDA receptor antagonist attenuated all effects of Glu. 

VlPAG has been revealed to be involved in cardiovascular regulation (11). Type of vlPAG projections is unknown, as some reports show that stimulation of vlPAG causes excitatory output from vlPAG (7). Despite that, some recent studies have shown that chemical stimulation of vlPAG causes depressor responses and a decrease in the heart rate (26, 27). It seems to contradict our findings, since Glu microinjection into vlPAG has shown pressor responses. One hypothesis is that the hypotensive effect of vlPAG was not mediated by Glu. Another possible suggestion is attributed to the consciousness condition, as we evaluated anesthetized rats, while that finding might be due to different conditions or methods, for instance, in unanesthetized decerebrate animals (28).

Ionotropic receptors of Glu are subdivided into two groups: NMDA and non-NMDA (AMPA and kainate) receptors, which affect several brain functions such as the learning process (29), control neuronal excitability (30), neural plasticity (31), and also autonomic responses (32). The central cardiovascular regulation of the NMDA receptors was also revealed in several brain regions such as RVLM, paraventricular nucleus (PVN), PBN, NTS, and vlPAG. These studies have reported that the Glu via NMDA and non-NMDA receptors can significantly increase the cardiovascular parameters in the mentioned nuclei (33-36). The observed results were also in line with the findings of these studies and showed that microinjection of the Glu into vlPAG significantly increased the cardiovascular responses in the normotensive rats. In this condition, the antagonist of the non-NMDA receptor in vlPAG had no remarkable effect on BP. The presence of both NMDA and non-NMDA receptors has been reported in vlPAG (14). It is known that Glu can activate all types of ionotropic-Glu receptors (37). Since the non-NMDA receptor in vlPAG did not demonstrate a considerable effect on cardiovascular regulation, it is suggested that NMDA was an essential receptor in vlPAG, which was involved in cardiovascular regulation. Consistent with these results, we also reported in our previous study that the NMDA receptor was the primary receptor in cardiovascular regulation in the CnF (20).

The excitatory effects of NMDA receptors are mediated by Glu and glycine receptor binding and channel permeability to Ca^2+^ conductance (38). Therefore, it was suggested that Glu in vlPAG had an excitatory effect on the cardiovascular system by the mentioned mechanism. On the other hand, it is shown that there are different neuron populations in vlPAG, including glutamatergic, GABAergic, dopaminergic, and serotonergic neurons (39). Since MK microinjection into vlPAG decreased BP and HR, it was inferred that Glu was released in normal conditions and activated an excitatory projection in vlPAG via NMDA receptor and increased the cardiovascular responses. 

vlPAG is a mesencephalic nucleus that has a great connection with the other brain regions associated with cardiovascular regulation, such as the caudal midline medulla (CMM), RVLM, CVLM, NTS, and the Cuneiform nucleus (CnF) (18, 40). So, it is reasonable that the cardiovascular effect of Glu is mediated via connection of vlPAG with the aforementioned areas. There is evidence that some of the pathways associated with the cardiovascular impact of vlPAG are indirect and mostly mediated via RVLM, which is a considerable important sympathoexcitatory region in the medulla (41).

Arterial pressure and vasoconstrictor tone are associated with sympathetic pathways to the heart and arteries, and it is known that the destination of the preganglionic sympathetic neurons is the intermediolateral column (IML) which has an essential role in the mediation of vlPAG-evoked cardiovascular responses (28). Since the direct projections from vlPAG to IML are unknown, it indicates indirect reaches to the IML through synapses in the pons or the medulla (28).

VlPAG-CVLM-RVLM pathway is reported (40), and it is known that there are glutamatergic projections from vlPAG (14) to GABAergic neurons in the CVLM projecting to RVLM (40). In the caudal medulla, there are probably many more than six distinct areas which excitatory amino acids (EAA) microinjection stimulate alteration (decrease or increase) in arterial blood pressure (40). The pressor effect induced by Glu microinjection into vlPAG could be mediated by the interaction of Glu and GABAergic neurons in the CVLM, as Glu causes disinhibition in GABAergic neurons of CVLM, so RVLM would not be affected by GABA, and therefore RVLM activation causes pressor responses. According to this evidence, it was suggested that Glu in vlPAG could decrease the activity of RVLM vasomotor neurons via disinhibition of GABAergic projections to RVLM.

VlPAG-CMM-RVLM pathway has been described as involved in cardiovascular regulation through glutamatergic projections to CMM (14, 42). It is known that there is a GABAergic-glutamatergic neural circuit in vlPAG, and activation or inhibition of each neural group can affect Glu neurons projecting to RVLM (43). CMM encompasses caudal raphe nuclei that its serotonergic neurons project to RVLM (41).

Due to the involvement of CMM and RVLM in cardiovascular regulation (40, 44), it was suggested that microinjecting Glu into vlPAG caused the interaction of Glu in vlPAG and serotoninergic neurons of CMM. So, it gives rise to disinhibition of pressor responses of RVLM. Although, the exact neurotransmitters involved are not clear and more studies are prerequisites for proving these suggestions. 

It is well known that raphe nuclei are involved in cardiovascular responses (45). Moreover, the excitatory afferents from vlPAG to nucleus raphe magnus (NRM) (46) and the rostral half of the nucleus raphe obscurus (NRO) are indicated (47). Mediation of vlPAG-raphe nuclei occurs with Glu (48). Hence, it is likely that glutamatergic projections from vlPAG could regulate cardiovascular activation via NRM or NRO. On the other hand, it has been shown that stimulation of these nuclei evokes inhibitory neurons projecting to RVLM (47). The possible hypothesis is that NRM/NRO-RVLM pathways are not monosynaptic, and RVLM stimulation is mediated indirectly via inhibitory interneurons. Therefore, activation of NRM or NRO neurons gives rise to disinhibition of RVLM and increasing BP.

VlPAG also projects to NTS, the region for integrating baroreceptor and chemoreceptor afferents (28). The cardiovascular responses of NMDA and non-NMDA receptors in the NTS have been reported (49). Hence, it is most likely that glutamatergic projections from vlPAG to the ionotropic receptors present in the NTS have a role in the HR regulation via baroreflex. 

In the rest of our experiment, we evaluate the role of NMDA and non-NMDA receptors of vlPAG during Hem. In the Hem condition, Glu reversed hypotension induced by Hem and enhanced HR. Moreover, blockade of the Glu receptors through MK decreased the cardiovascular responses induced by Hem. Thus, it shows the role of the NMDA receptor in mediating the hemodynamic responses during Hem. 

The role of NMDA and non-NMDA receptors in cardiovascular regulation during Hem condition is indicated (50). For the first time, we evaluated Hem-induced hemodynamic responses via ionotropic Glu receptors of vlPAG. 

There is comprised of the effect of the glutamatergic system in Hem (51). Research points out that NMDA receptors are involved in Hem (52). It is assumed that the effect of Glu might be accompanied by other neurotransmitters such as glycine, norepinephrine, serotonin, acetylcholine, and GABA (53). These neurotransmitters alter the sympathetic activity during Hem in cardiovascular regions at the medulla level, including RVLM, NTS, and CVLM (53).

In line with the current study, another study revealed that mu receptor agonist microinjection into vlPAG reversed hemodynamic reflexes, followed by Hem (54). According to vlPAG and RVLM connections, it seems that the Glu pathway from vlPAG to RVLM had an excitatory effect on the cardiovascular reflexes induced by Hem. It is well documented that there is a GABAergic-Gluergic neural circuit in vlPAG, and activation or inhibition of each neural group can affect Glu neurons projecting to RVLM (43).

The serotonergic neurons of raphe nuclei are involved in the cardiovascular reflexes following Hem (55). Also, 5-HT_1A_ receptors in RVLM participate in sympathoinhibitory responses during Hem (56). As mentioned earlier, there is a correlation between vlPAG and midline raphe nuclei (47). In addition, serotonergic neurons are one of the different neurons population in vlPAG (39). Thus, Glu’s possible mechanism attenuating hypotensive responses in Hem is that activated serotonergic neurons of vlPAG inhibit serotonergic neurons of raphe nuclei and cause disinhibition in RVLM. The raphe nuclei affect BP and HR via innervating preganglionic sympathetic neurons in the spinal cord, directly or via indirect projections to the CVLM or RVLM (54). Hence, it is possible that the Glu receptors participated in cardiovascular regulation during Hem through interaction with serotonin receptors of raphe nuclei.

VlPAG also projects arising neurons to the PVN (57). Also, PVN is involved in response to blood volume reduction via vasopressin secretion (58); therefore, it is possible that activating the Glu receptors in vlPAG via increase of vasopressin release from PVN also enhanced the cardiovascular parameters. Although, this hypothesis requires future experiments to prove it.

In the current study, the tachycardia due to blood withdrawal, enhanced by Glu microinjection. The mechanism of this impact is poorly documented. Though, baroreflex activates following Hem and maintains BP and HR close to the baseline. The presence of both NMDA and non-NMDA receptors has been indicated in the NTS that take part in the baroreflex (59, 60). Since the NMDA receptor antagonist in vlPAG attenuated HR, the mentioned receptor likely participates in HR regulation via NTS.

## Conclusion

The present study revealed that activation of NMDA receptors of vlPAG enhanced the cardiovascular responses in normotensive and hemorrhagic hypotensive rats. Considering the inhibitory role of vlPAG, it seems the Glu does not have an important role in normotensive and hemorrhagic conditions. Concerning this neurotransmitter nature, it has a stimulatory effect.

## References

[1] Lagatta DC, Ferreira-Junior NC, Deolindo M, Corrêa FM, Resstel LB (2016). Ventrolateral periaqueductal grey matter neurotransmission modulates cardiac baroreflex activity. Eur J Neuro Sci.

[2] Dampney R (2018). Emotion and the cardiovascular system: postulated role of inputs from the medial prefrontal cortex to the dorsolateral periaqueductal gray. Front Neurosci.

[3] Wright KM, Jhou TC, Pimpinelli D, McDannald MA (2019). Cue-inhibited ventrolateral periaqueductal gray neurons signal fear output and threat probability in male rats. Elife.

[4] Hao S, Yang H, Wang X, He Y, Xu H, Wu X (2019). The lateral hypothalamic and BNST GABAergic projections to the anterior ventrolateral periaqueductal gray regulate feeding. Cell Rep.

[5] Kroeger D, Bandaru SS, Madara JC, Vetrivelan R (2019). Ventrolateral periaqueductal gray mediates rapid eye movement sleep regulation by melanin-concentrating hormone neurons. Neuroscience.

[6] Sun Y, Wang J, Liang S-H, Ge J, Lu Y-C, Li J-N (2020). Involvement of the ventrolateral periaqueductal gray matter-central medial thalamic nucleus-basolateral amygdala pathway in neuropathic pain regulation of rats. Front Neuroanat.

[7] Tjen-A-Looi SC, Li P, Longhurst JC (2006). Midbrain vlPAG inhibits rVLM cardiovascular sympathoexcitatory responses during electroacupuncture. Am J Physiol Heart Circ.

[8] Barbosa RM, Speretta GF, Dias DPM, Ruchaya PJ, Li H, Menani JV (2017). Increased expression of macrophage migration inhibitory factor in the nucleus of the solitary tract attenuates renovascular hypertension in rats. Am J Hypertens.

[9] Vagg DJ, Bandler R, Keay KA (2008). Hypovolemic shock: critical involvement of a projection from the ventrolateral periaqueductal gray to the caudal midline medulla. Neuroscience.

[10] Shafei MN, Nasimi A, Alaei H, Pourshanazari AA (2009). The role of non-NMDA receptor of glutamate in cuneiform nucleus on cardiovascular response in anaesthetized rats. Pharmacology Online.

[11] Deolindo M, Pelosi GG, Tavares RF, Corrêa FMA (2008). The ventrolateral periaqueductal gray is involved in the cardiovascular response evoked by l-glutamate microinjection into the lateral hypothalamus of anesthetized rats. Neurosci Lett.

[12] Takahashi M, Hayashi Y, Tanaka J (2017). Glutamatergic modulation of noradrenaline release in the rat median preoptic area. Brain Res Bull.

[13] Yamaguchi Ki, Yamada T (2006). Involvement of anteroventral third ventricular AMPA/kainate receptors in both hyperosmotic and hypovolemic AVP secretion in conscious rats. Brain Res Bull.

[14] Samineni VK, Grajales-Reyes JG, Copits BA, O’Brien DE, Trigg SL, Gomez AM (2017). Divergent modulation of nociception by glutamatergic and GABAergic neuronal subpopulations in the periaqueductal gray. eNeuro.

[15] Pajolla GP, de Aguiar Corrêa FM (2004). Cardiovascular responses to the injection of L-glutamate in the lateral hypothalamus of unanesthetized or anesthetized rats. Auton Neurosci.

[16] Yang Y, Lu F, Zhuang L, Yang S, Kong Y, Tan W (2017). Combined preconditioning with hypoxia and GYKI-52466 protects rats from cerebral ischemic injury by HIF-1α/eNOS pathway. Am J Transl Res..

[17] Okada M, Fukuyama K, Nakano T, Ueda Y (2019). Pharmacological discrimination of effects of MK801 on thalamocortical, mesothalamic, and mesocortical transmissions. Biomolecules..

[18] Mohebbati R, Hosseini M, Khazaei M, Khajavirad A, Shafei MN (2020). Involvement of the 5-HT1A receptor of the cuneiform nucleus in the regulation of cardiovascular responses during normal and hemorrhagic conditions. Iran J Basic Med Sci.

[19] Paxinos G, Watson C (2009). The rat brain in stereotaxic coordinates: compact sixth edition. New York: Academic Press.

[20] Shafei MN, Nasimi A (2011). Effect of glutamate stimulation of the cuneiform nucleus on cardiovascular regulation in anesthetized rats: Role of the pontine Kolliker–Fuse nucleus. Brain Res.

[21] Martin DS, Haywood JR (1992). Sympathetic nervous system activation by glutamate injections into the paraventricular nucleus. Brain Res.

[22] Geambasu A, Krukoff TL (2008). Adrenomedullin acts in the lateral parabrachial nucleus to increase arterial blood pressure through mechanisms mediated by glutamate and nitric oxide. Am J Physiol Regu..

[23] Donevan SD, Rogawski MA (1993). GYKI 52466, a 2, 3-benzodiazepine, is a highly selective, noncompetitive antagonist of AMPA/kainate receptor responses. Neuron.

[24] Ahlgren J, Porter K, Hayward LF (2007). Hemodynamic responses and c-Fos changes associated with hypotensive hemorrhage: standardizing a protocol for severe hemorrhage in conscious rats. Am J Physiol Regul.

[25] Shafei MN, Nasimi A, Alaei H, Pourshanazari AA, Hosseini M (2012). Role of cuneiform nucleus in regulation of sympathetic vasomotor tone in rats. Pathophysiology.

[26] Lagatta DC, Ferreira-Junior NC, Deolindo M, Corrêa FM, Resstel LB (2016). Ventrolateral periaqueductal grey matter neurotransmission modulates cardiac baroreflex activity. Eur J Neurosci.

[27] Deolindo MV, Pelosi GG, Busnardo C, Resstel LB, Corrêa FM (2011). Cardiovascular effects of acetylcholine microinjection into the ventrolateral and dorsal periaqueductal gray of rats. Brain Res.

[28] Depaulis A, Bandler R The midbrain periaqueductal gray matter: Functional, anatomical, and neurochemical organization. Springer Science & Business Media.

[29] Riedel G, Platt B, Micheau J (2003). Glutamate receptor function in learning and memory. Behav. Brain Res.

[30] Altevogt BM, Davis M, Pankevich DE (2011). Glutamate-Related biomarkers in drug development for disorders of the nervous system: Workshop summary. National Academies Press.

[31] Nakanishi S, Nakajima Y, Masu M, Ueda Y, Nakahara K, Watanabe D (1998). Glutamate receptors: Brain function and signal transduction. Brain Res Rev.

[32] Bereiter DA (1993). Microinjections of glutamate within trigeminal subnucleus interpolaris alters adrenal and autonomic function in the cat. Brain Res.

[33] Len W-B, Chan SH, Chan JY (2000). Parabrachial nucleus induces suppression of baroreflex bradycardia by the release of glutamate in the rostral ventrolateral medulla of the rat. J Biomed Sci.

[34] Guyenet PG (2006). The sympathetic control of blood pressure. Nat Rev Neurosci.

[35] Zoccal DB, Furuya WI, Bassi M, Colombari DS, Colombari E (2014). The nucleus of the solitary tract and the coordination of respiratory and sympathetic activities. Front Physiol.

[36] Sweazey RD (1995). Distribution of aspartate and glutamate in the nucleus of the solitary tract of the lamb. Exp Brain Res.

[37] Nasimi A, Shafei M, Alaei H (2012). Glutamate injection into the cuneiform nucleus in rat, produces correlated single unit activities in the Kolliker-Fuse nucleus and cardiovascular responses. Neuroscience.

[38] Tu Y-C, Yang Y-C, Kuo C-C (2016). Modulation of NMDA channel gating by Ca 2+ and Cd 2+ binding to the external pore mouth. Sci Rep.

[39] Taylor NE, Pei J, Zhang J, Vlasov KY, Davis T, Taylor E (2019). The role of glutamatergic and dopaminergic neurons in the periaqueductal gray/Dorsal raphe: separating analgesia and anxiety. Eneuro.

[40] Henderson L, Keay K, Bandler R (1998). The ventrolateral periaqueductal gray projects to caudal brainstem depressor regions: a functional-anatomical and physiological study. Neuroscience.

[41] Dean C (2005). Sympathoinhibition from ventrolateral periaqueductal gray mediated by the caudal midline medulla. Am J Physiol Regul Integr Comp Physiol.

[42] Dean C (2004). Hemorrhagic sympathoinhibition mediated through the periaqueductal gray in the rat. Neurosci Lett.

[43] Zhu H, Xiang H-C, Li H-P, Lin L-X, Hu X-F, Zhang H (2019). Inhibition of GABAergic neurons and excitation of glutamatergic neurons in the ventrolateral periaqueductal gray participate in electroacupuncture analgesia mediated by cannabinoid receptor. Front Neurol.

[44] Guyenet PG, Stornetta RL, Holloway BB, Souza GM, Abbott SB (2018). Rostral ventrolateral medulla and hypertension. Hypertension.

[45] Berthoud H-R, Patterson LM, Sutton GM, Morrison C, Zheng H (2005). Orexin inputs to caudal raphe neurons involved in thermal, cardiovascular, and gastrointestinal regulation. Histochem Cell Biol.

[46] Wiklund L, Behzadi G, Kalén P, Headley PM, Nicolopoulos LS, Parsons CG (1988). Autoradiographic and electrophysiological evidence for excitatory amino acid transmission in the periaqueductal gray projection to nucleus raphe magnus in the rat. Neurosci Lett.

[47] Wang W, Lovick T (1993). The inhibitory effect of the ventrolateral periaqueductal grey matter on neurones in the rostral ventrolateral medulla involves a relay in the medullary raphe nuclei. Exp. Brain Res.

[48] Mokhtar M, Singh P (2020). Neuroanatomy, periaqueductal gray. StatPearls [Internet]: StatPearls Publishing.

[49] Talman W (1997). Glutamatergic transmission in the nucleus tractus solitarii: from server to peripherals in the cardiovascular information superhighway. Braz J Med Biol Res.

[50] Busnardo C, Crestani C, Fassini A, Resstel L, Corrêa F (2016). NMDA and non-NMDA glutamate receptors in the paraventricular nucleus of the hypothalamus modulate different stages of hemorrhage-evoked cardiovascular responses in rats. Neuroscience.

[51] Dávalos A, Shuaib A, Wahlgren NG (2000). Neurotransmitters and pathophysiology of stroke: Evidence for the release of glutamate and other transmitters/mediators in animals and humans. J Stroke Cerebrovasc Dis.

[52] Yamaguchi Ki, Watanabe K (2005). Anteroventral third ventricular N-methyl-D-aspartate receptors, but not metabotropic glutamate receptors are involved in hemorrhagic AVP secretion. Brain Res Bull.

[53] Takemoto Y (2012). Amino acids that centrally influence blood pressure and regional blood flow in conscious rats. J Amino Acids.

[54] Cavun S, Resch GE, Evec AD, Rapacon-Baker MM, Millington WR (2001). Blockade of delta opioid receptors in the ventrolateral periaqueductal gray region inhibits the fall in arterial pressure evoked by hemorrhage. J Pharmacol Exp Ther.

[55] Kung L-H, Glasgow J, Ruszaj A, Gray T, Scrogin KE (2010). Serotonin neurons of the caudal raphe nuclei contribute to sympathetic recovery following hypotensive hemorrhage. Am J Physiol Regul Integr Comp Physiol.

[56] Dean C, Bago M (2002). Renal sympathoinhibition mediated by 5-HT1Areceptors in the RVLM during severe hemorrhage in rats. Am J Physiol Regul Integr Comp Physiol.

[57] Floyd NS, Keay KA, Arias CM, Sawchenko PE, Bandler R (1996). Projections from the ventrolateral periaqueductal gray to endocrine regulatory subdivisions of the paraventricular nucleus of the hypothalamus in the rat. Neurosci Lett.

[58] Yang Z, Coote J (2007). Paraventricular nucleus influence on renal sympathetic activity in vasopressin gene-deleted rats. Exp Physiol.

[59] Li C-S, Smith DV (1997). Glutamate Receptor Antagonists block gustatory afferent input to the nucleus of the solitary tract. J Neurophysiol.

[60] Ohta H, Talman WT (1994). Both NMDA and non-NMDA receptors in the NTS participate in the baroreceptor reflex in rats. Am J Physiol Regul Integr Comp Physiol.

